# dsRNA Uptake in Plant Pests and Pathogens: Insights into RNAi-Based Insect and Fungal Control Technology

**DOI:** 10.3390/plants9121780

**Published:** 2020-12-16

**Authors:** Nick Wytinck, Christopher L. Manchur, Vivian H. Li, Steve Whyard, Mark F. Belmonte

**Affiliations:** Department of Biological Sciences, University of Manitoba, Winnipeg, MB R3T 2N2, Canada; wytinckn@myumanitoba.ca (N.W.); manchurc@myumanitoba.ca (C.L.M.); liv1@myumanitoba.ca (V.H.L.); Steve.Whyard@umanitoba.ca (S.W.)

**Keywords:** clathrin-mediated endocytosis, double stranded RNA, dsRNA uptake, fungi, insects, plant protection, RNA interference, SID proteins, spray induced gene silencing

## Abstract

Efforts to develop more environmentally friendly alternatives to traditional broad-spectrum pesticides in agriculture have recently turned to RNA interference (RNAi) technology. With the built-in, sequence-specific knockdown of gene targets following delivery of double-stranded RNA (dsRNA), RNAi offers the promise of controlling pests and pathogens without adversely affecting non-target species. Significant advances in the efficacy of this technology have been observed in a wide range of species, including many insect pests and fungal pathogens. Two different dsRNA application methods are being developed. First, host induced gene silencing (HIGS) harnesses dsRNA production through the thoughtful and precise engineering of transgenic plants and second, spray induced gene silencing (SIGS) that uses surface applications of a topically applied dsRNA molecule. Regardless of the dsRNA delivery method, one aspect that is critical to the success of RNAi is the ability of the target organism to internalize the dsRNA and take advantage of the host RNAi cellular machinery. The efficiency of dsRNA uptake mechanisms varies across species, and in some uptake is negligible, rendering them effectively resistant to this new generation of control technologies. If RNAi-based methods of control are to be used widely, it is critically important to understand the mechanisms underpinning dsRNA uptake. Understanding dsRNA uptake mechanisms will also provide insight into the design and formulation of dsRNAs for improved delivery and provide clues into the development of potential host resistance to these technologies.

## 1. Introduction

Each year, crop pests and pathogens cause approximately 300 billion USD of damage to plant-based food supplies worldwide [1]. For five of the major food crops (rice, wheat, maize, soybean, and potato), 17–30% of annual global yield losses can be directly attributed to these biotic factors [2]. Latest projections suggest that, by 2050, we will need to increase food production by more than 50% to feed a population that will be nearing 10 billion people [3]. Furthermore, as agriculture has shifted to an intensive, monoculture state to accommodate rising demand, this has favoured the occurrence of widespread epidemics and outbreaks from pests and pathogens [4]. Climate change also creates additional stresses on land suitable for food production. The frequency and intensity of droughts is expected to increase, promoting further desertification, particularly in Africa and Asia [5]. Rising sea levels also contribute to soil erosion and increased salinity and increased extreme weather events such as floods and cyclones will also reduce arable land mass [6]. The challenges posed by pests and pathogens, a rapidly growing global population, and unpredictable climactic conditions demand that we find new and innovative solutions to maintain healthy crops without losing yield. 

Current approaches to manage insect pests and to minimize damage relies on the use of broad-spectrum chemical pesticides such as neonicotinoids, organophosphates, carbamates and pyrethroids. Each year an average of 1 billion pounds of active ingredient is applied globally to crops such as corn, cotton, fruits and vegetables to control these insect pests [7]. Although improvements have been made in terms of environmental toxicity compared to early pesticide compounds such as dichloro-diphenyl-trichloroethane (DDT), concerns remain for existing chemistries relating to environmental dispersal and persistence causing lethal non-target effects [8]. Evidence suggests populations of beneficial arthropods such as pollinators and aquatic invertebrates have been harmed by the presence of these traditional chemistries leading to several nations placing legislation against the use of the certain classes of chemicals [9,10,11]. 

Similar to insect pest control, the control of phytopathogenic fungi relies heavily on broad-spectrum chemistries. Commonly used fungicidal classes include mitosis disruptors (methyl benzimidazole carbamates), cell membrane disruptors (triazoles), and respiration inhibitors (strobilurins) [12,13,14]. Like insecticides, unintentional off-target effects present a problem to traditional fungal control technologies. Fungicides have been shown to adversely affect insect species, especially key pollinators such as bees, aquatic species from chemical runoff into waterways, and beneficial soil microorganisms [15,16,17,18,19,20,21,22]. In some cases, broad-spectrum chemistries targeting either fungi or insects are becoming less effective as resistance to these chemicals evolves [23,24,25,26]. Taken together, an environmentally safe alternative that poses less risk to the agroecological environment may provide a solution to improve crop health.

One alternative to chemical pesticides with the potential for species-specificity is RNA interference (RNAi). RNAi has been observed in a wide range of eukaryotic organisms and has emerged as a powerful tool to study gene function [27]. RNAi mediates RNA destruction following the introduction of dsRNA molecules, thereby reducing the expression of a target gene. Data show that synthetic dsRNA can be used to target and knockdown specific genes within an organism [28,29,30,31]. In insects, the order Coleoptera is highly sensitive to RNAi [32,33], however the Diptera [34,35] Hemiptera [36,37], and Lepidoptera [38,39,40,41] have shown varying levels of sensitivity. In fungi, most species contain the enzymatic machinery necessary for RNAi and gene knockdown is achievable with a few exceptions, including *Saccharomyces cerevisiae* and *Ustilago maydis* [42]. Researchers have harnessed and applied this technology to control agricultural pests by designing dsRNAs targeting essential genes and thus are able to disrupt cellular functions thus impairing or killing the target species [31,43,44,45]. The uptake of dsRNA targeting essential genes by insect and fungal pests can lead to growth inhibition, reduced pathogenicity and mortality. Unlike chemical pesticides, which may affect a broad range of species, RNAi is sequence specific and can target a single species, leaving other beneficial organisms unharmed. Seminal studies of RNAi-based pest control employed a strategy known as host induced gene silencing (HIGS), where the host plant is engineered to express the dsRNA molecules for insect or fungal protection [44,46,47]. For insects, the dsRNA molecules are absorbed through intestinal uptake following feeding, allowing for systemic spread. Currently, the only commercialized example of HIGS technology is in maize and targets the vacuolar sorting protein Snf7 of the western corn rootworm (*Diabrotica virgifera*). 

While HIGS offers the promise of long-lasting protection, RNAi can also be used as a topical formulation to avoid difficulties associated with plant transformation and the regulation of genetically modified organisms in different markets [48]. Spray induced gene silencing (SIGS) involves the foliar applications of dsRNA to the plant surface. A number of studies in both fungi and insects have demonstrated the effectiveness of this technology [31,45,49,50]. Unlike many chemical pesticides, environmental persistence of dsRNAs appears to be limited, which, from an environmental protection perspective, is an attractive feature [51,52]. Studies have shown a half-life of less than 24 h for dsRNA within the soil, due primarily to bacterial degradation [51]. However, they are stable within the phyllosphere, where they can remain biologically active for several weeks [53]. Unlike transgenic approaches, spray-based control methods are more appropriate for the control of pests or pathogens that affect multiple crops. For example, SIGS does not require the development and approval of genetically engineered technologies for each crop species and does not limit the technology to single gene or application [54]. Due to the large number of coding genes within organisms, this presents the opportunity to design dsRNAs to multiple targets. In several studies, multiple gene targets were shown to be effective when dsRNAs were applied as a foliar treatment, thus providing insurance and allowing for alteration of targets between growing seasons [45,55,56]. 

This review will explore proposed mechanisms of dsRNA uptake in eukaryotes as a means to control both insect pests and fungal pathogens through RNAi. SIGS provides considerable promise, both in terms of offering a new generation of pesticides that are environmentally more benign than most current pesticides, and in terms of applying RNA technologies in a delivery method that avoids the challenges surrounding the regulation of genetic modification. Understanding the uptake of dsRNAs through SIGS will help accelerate the development, implementation, and application of this technology outside of the laboratory or greenhouse environment. While recent studies clearly demonstrate the potential of SIGS as a tool to control insect pests and fungal pathogens, we still require a deeper understanding of target species that are sensitive or those refractory to RNAi. The current review updates our understanding of RNAi and provides novel insight into the requirements necessary to develop successful alternatives to exogenously-applied broad-spectrum chemistries. Specifically, we provide strategies to improve dsRNA uptake through the optimization and development of exogenously-applied dsRNA formulations and delivery methods. 

## 2. Core Components of the RNAi Machinery

Since the first description of sequence-specific gene silencing in the nematode *Caenorhabditis elegans* in 1998 [57], RNAi has been well documented in almost all eukaryotic organisms, including protozoans, invertebrates, vertebrates, plants, fungi, and algae. Before the term RNAi was widely adopted, RNA silencing had been described as post transcriptional gene silencing in plants and quelling in fungi [58,59], but each of these different names refer to a common process, with shared intracellular machinery. The core components of RNAi have now been identified in all major branches of eukaryotes. 

The protein Dicer is the initiator of the RNAi pathway. Dicer belongs to the RNase III family, an evolutionarily conserved protein group with specificity for dsRNAs and is responsible for processing long dsRNAs into smaller duplex fragments of discrete sizes [60]. While Dicer is found in virtually all eukaryotes, it has diversified structurally as well as functionally, producing several types of small RNA [61]. Dicers are essential for the biogenesis of both small interfering (si)RNA and micro (mi)RNA, however only siRNAs are involved in the targeting of exogenous mRNA while miRNAs play a regulatory role [60]. The Dicer generated siRNAs are subsequently co-opted by the RNAi-induced silencing complex (RISC), which unwinds the siRNA duplex. The exact molecular composition of RISC has yet to be defined but must at least contain an Argonaute protein [62]. Argonaute cleaves the passenger (sense) strand of the siRNA while the guide (antisense) strand remains connected with the RISC [63]. The guide strand of the siRNA within RISC to base pairs with complementary target mRNAs, which are then cleaved by Argonaute, thereby preventing translation [64]. Some species also possess RNA-dependent RNA polymerases (RdRPs), which catalyze the replication of dsRNA from aberrant single stranded-RNA transcripts [65]. RdRPs function to amplify the RNA silencing signal, thus promoting systemic spread. 

While many of the core RNAi components are conserved throughout taxa, RNAi efficiency varies across species, and many of the variations are due to differences in the efficiency of dsRNA uptake, intracellular distribution, and/or systemic dispersal. For the remainder of this review, the different modes of uptake and dsRNA movements will be examined, highlighting where there are gaps in our understanding, and how these should be addressed if RNAi technologies are to be deployed more widely.

## 3. Mechanisms of dsRNA Uptake

### 3.1. Caenorhabditis elegans: Systemic RNAi Defective (SID) Proteins

In addition to the discovery of RNAi, the mechanism of dsRNA uptake and cell to cell spread (systemic RNAi) was also first described in *C. elegans*. Soaking nematodes in dsRNA or feeding them bacteria producing dsRNA induces RNAi both within the intestine and in tissues distal from the site of ingestion. This systemic RNAi in nematodes [66] is mediated by multiple SID proteins that facilitate the transfer of dsRNAs or siRNAs throughout the body. SID-1, a dsRNA specific membrane channel, has been studied extensively [67,68,69]. *Sid-1* mutants are insensitive to RNAi and unable to spread the silencing signal to adjacent cells. However, silencing can still be achieved through cell autonomous RNAi using a direct injection delivery or by transgenic expression [67]. Additionally, there does not appear to be selective uptake based on dsRNA length through the channel [70].

SID-2 is a single pass transmembrane protein located specifically in the luminal membranes of the intestine and is involved in dsRNA uptake within the gut of *C. elegans*. SID-2 is thought to mediate uptake through interaction and delivery of the dsRNA to SID-1 or through endocytosis, with SID-1 thereafter enabling the dsRNA to escape the endosome and enter the cytoplasm [71,72]. SID-3, like SID-1, is also required for efficient import of dsRNA [73]. SID-3 is ubiquitously expressed in most tissue types and is important in the uptake of dsRNA in the recipient cell. In addition to being associated with endocytic vesicles, SID-3 appears to be an ortholog of ACK tyrosine kinase although its phosphorylation targets and its interaction with SID-1 have yet to be described [73]. 

Another SID protein, SID-5, has been shown to be required for extracellular spreading of the RNAi signal [74]. SID-5 is predicted to have a single transmembrane domain and interacts with late endosomal proteins such as RAB7 [75]. Since Rab7 GTPases are responsible for the regulation of late endosomal trafficking into lysosomes, Hinas et al. [74] proposed that SID-5 functions to block exosomal fusion with lysosomes and allows for exosomal release from the cell. The complex roles and interactions of the multiple SID proteins in *C. elegans* still need to be fully resolved, but it is clear that endocytic pathways are integral to the uptake and dispersal of dsRNAs in this species. 

### 3.2. Insects: SID-Like Proteins 

Orthologous proteins to *C. elegans* SID-1, called SID-like (SIL-A, SIL-B, and SIL-C), have been identified in several insect species, although their direct involvement in dsRNA uptake has yet to be determined in many cases [76]. In cotton and soybean aphids (*Aphis gossypii, Aphis glycines*), for example, the SIL proteins are structurally similar to *C. elegans* SID-1, [77,78], but their role in mediating dsRNA uptake has not been confirmed. In the honeybee (*Apis mellifera*), expression of a SIL gene increased following exposure to dsRNA, suggesting a role in mediating RNAi, but again, the role of the encoded protein in dsRNA uptake was not determined [79]. Reduced RNAi efficiencies were observed in the Western corn rootworm *(D. virgifera)* and Colorado potato beetle (*Leptinotarsa decemlineata)* following knockdown of the SIL mRNAs, indicating at least a partial role of SIL proteins in modulating RNAi in these two beetles [80,81]. In contrast, the flour beetle *Tribolium castaneum* has three orthologs of SID-1, but when all three were silenced, there was no effect on RNAi efficiency [82]. Similarly, the SIL proteins in the desert locust (*Schistocerca gregaria)* or diamondback moth (*Plutella xylostella*) appear to play no role in RNA efficiency [83,84]. In dipteran insects, no SIL orthologues have been found, and yet RNAi has been demonstrated in many flies and mosquitoes [85,86,87], indicating that SIL proteins are likewise not required for dsRNA uptake in these insects. Based on the range of insects studied thus far, the role of SIL proteins in dsRNA uptake in these organisms is clearly variable, with SIL facilitating RNAi in some species, but not in others. 

Reverse BLAST searches between insect SIL proteins and the *C. elegans* proteome suggest that SIL proteins more closely resemble TAG-I30/CHUP-1 proteins than SID-1 [82]. CHUP-1 genes have no apparent role in uptake or systemic RNAi within the nematode and when *C. elegans* CHUP-1 genes were transfected into *Drosophila* S2 cells, no dsRNA uptake was observed [69]. CHUP-1 has a role in the cellular uptake of cholesterol [88], and there is some speculation that a depletion of cholesterol levels may perturb or disrupt clathrin-mediated endocytosis and vesicle transport, possibly affecting the amount of dsRNA that enters the cell [89]. Moreover, the efficacy of dsRNA uptake may be influenced by the fatty acid composition of the cellular membrane. The ratio of the poly-unsaturated fatty acids, linoleic acid, and arachidonic acid in the membrane was shown to be important, and the injection of arachidonic acid improved the RNAi response in *Bactrocera dorsalis* [90].

### 3.3. Insects: Endocytosis

Uptake of dsRNAs in several insect species has been shown to involve clathrin-mediated endocytosis. The involvement of endocytosis was first described in S2 *Drosophila* cells, which lack any SID-like proteins. through experiments providing both direct and indirect evidence of this process [91,92]. Accumulation of fluorescently labelled dsRNA within distinct cytoplasmic vesicles of S2 cells provided direct evidence of uptake, and pretreatment of cells with inhibitors of endocytic processes such as chlorpromazine and bafilomycin A1 indicated that clathrin-mediated endocytosis was facilitating the process [91,92]. RNAi-mediated knockdown of genes encoding proteins involved in clathrin dependent endocytosis, resulted in impaired RNAi of a secondary reporter gene, confirming that uptake of dsRNA was occurring by this process [91,92]. The proteins identified in the RNAi knockdown screens encompassed the entire endocytic pathway, from early vesicle formation (clathrin, AP50) to late endosomal release (Rab7 and Vacuolar H^+^ ATPase), indicating that the dsRNA was traversing through the endosomal pathway, only to be released to the cytoplasm before being degraded within the lysosomes. Knockdown of these same endocytic components in *C. elegans* also impaired subsequent RNAi, providing additional evidence that endocytosis is an important component of the dsRNA uptake process even in species that rely on SID proteins to support uptake [91].

**Figure 1:**
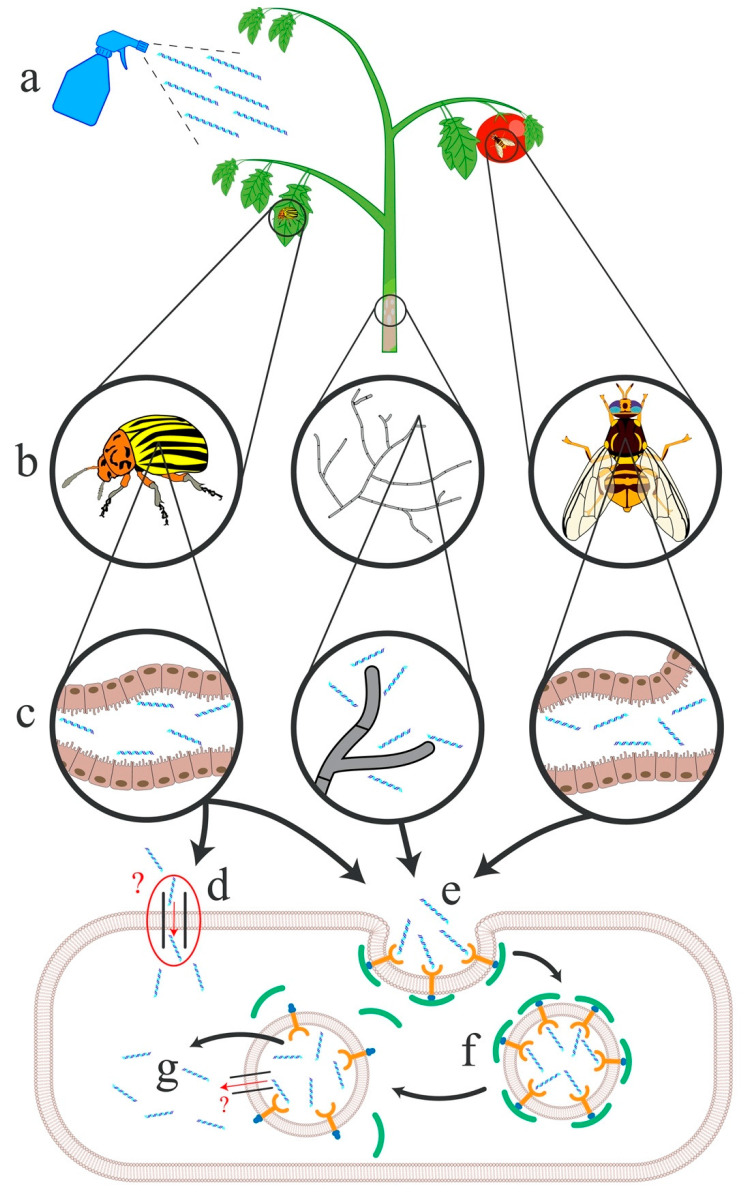
Proposed modes of dsRNA uptake within insect pests and fungal pathogens using *L. decemlineata, S. sclerotiorum* and *B. dorsalis* as representative species. (**a**) Spray application of dsRNA formulations; (**b**) dsRNAs are transferred to the insect pest or fungal pathogen upon (**c**) ingestion of plant material by the insect gut or absorption by fungal hyphae. dsRNAs are internalized by SIL proteins (**d**) in *L. decemlineata* or through clathrin-mediated endocytosis (**e**) in *L. decemlineata, S. sclerotiorum* and *B. dorsalis*. Following endosomal release and maturation (**f**), dsRNAs are released into the cytoplasm from the endosome (**g**), potentially facilitated by SIL proteins in *L. decemlineata.*

Clathrin-mediated endocytosis has been demonstrated to be involved in uptake of exogenous dsRNA in several more insect species, using labeled dsRNA to track cellular entry. In *T. castaneum,* fluorescent labeling and endocytic inhibitors provided direct evidence of clathrin-mediated endocytosis in uptake [93]. In *B. dorsalis* and *D. virgifera* clathrin related genes were identified through an RNAi mediated knockdown approach (Figure 1) [94,95]. Interestingly, in some species such as *L. decemlineata*, both SIL channels and endocytosis appear to be involved (Figure 1) [81]. Vacuolar H^+^ ATPase and the clathrin heavy chain were identified through RNAi mediated knockdown experiments, in addition to SIL-A and SIL-C. However, the relative contribution of each uptake mechanism remains uncertain. The endocytic process encompasses dsRNA binding to a receptor, inducing the invagination of the membrane. Clathrin and its adaptors are then recruited and a vesicle forms and releases from the membrane. The endosomal vesicle matures and via pH shifts from proton pumps, the dsRNA is released into the cytoplasm. It is still unknown when the dsRNA is released and how it is moved throughout the cells. It is interesting to note that in lepidopteran cells, which are generally more recalcitrant to RNAi, the dsRNA can enter the cells but remains trapped in the endosomes [96]. It is unclear what factors are preventing the dsRNA escape from the endosomal pathway, but for these species, efforts to improve RNAi efficiency have focused on alternative delivery molecules to help the dsRNA reach the cytoplasm (see dsRNA Formulation section). In contrast to SID proteins, length of the dsRNA does appear to play a role in endocytic-mediated uptake. For example, shorter molecules do not appear to enter through this transport system as efficiently as longer ones [97,98]. In *T. castaneum,* a 31 base-pair dsRNA fragment was unable to achieve successful knockdown while a 69 base-pair long fragment was able to reduce the accumulation of the target mRNA. The authors suggest that short dsRNAs are not efficiently recognized by the dsRNA uptake machinery and may not be incorporated into the cell thus preventing sufficient target knockdown [98].

Two *Drosophila* scavenger receptors, SR-CI and Eater were also identified in Ulvila’s [92] screen and were found to account for over 90% of dsRNA uptake in *Drosophila* S2 cells. These receptors are involved in receptor mediated phagocytosis of gram negative and positive bacteria in *Drosophila*. In the desert locust (*S. gregaria*) SR-CI and Eater were also identified to be important to uptake through chemical inhibition of the receptor [83]. However, only a weak response of knocking down another scavenger receptor, SC-R2, was observed in Colorado potato beetle cell lines [99]. In the *Aedes aegypti* mosquito cells, where clathrin-mediated endocytosis facilitated uptake, chemical inhibition of scavenger receptors had no impact on dsRNA uptake [100]. Based on these findings, it seems likely that dsRNAs can bind to different, and perhaps multiple receptors in each species. Determining the identity, structure, and selectivity of these receptors will prove valuable in the design of dsRNAs with improved binding affinities and uptake capabilities.

### 3.4. Fungal Uptake

Without orthologs to SID proteins, fungi also appear to rely on endocytosis to facilitate uptake of dsRNA. Uptake of fluorescently labelled dsRNA in fungi was first reported in *Botrytis cinerea* by Wang et al. [31], although the uptake mechanism was not identified. A study by Wytinck et al. [101] demonstrated that uptake of dsRNA in *Sclerotinia sclerotiorum* occurs through clathrin-mediated endocytosis, analogous to insect systems that do not rely on SIL channels (Figure 1). While a dsRNA specific receptor was not identified in the study, endocytic proteins CHC, Arf72A, AP2, FCHO1, amphiphysin, and VH^+^ ATPase were shown to be involved in dsRNA uptake and processing through RNAi mediated knockdown experiments. They demonstrated that uptake is localized to the hyphal tip in younger, more actively growing hyphae through live cell imaging. Endocytosis has also been shown to localize at the hyphal tip of the developing fungus [102]. 

Fungi lack homologues to the insect scavenger receptors, and therefore genome wide screens may be necessary in order to identify potential candidate dsRNA receptors. The list of candidates could then be narrowed to those that specifically bind dsRNA through affinity capture techniques. The role of the putative receptor in dsRNA uptake could then be assessed through RNAi mediated knockdown. Within the fungal kingdom, most fungi assessed are amenable to RNAi, however a couple such as *U. maydis* and *S. cerevisiae* lack the core RNAi components such as Dicer or Argonaute [103]. One notable exception is *Zymoseptoria tritici* which encodes the core components however is still insensitive to dsRNA [104]. Through live cell imaging, the authors showed that conidiospores of *Z. tritici* were unable to uptake dsRNA, suggesting that there may be not be a dsRNA receptor encoded or there is a defect in the uptake pathway. Overall, there is a dearth of information relating to dsRNA uptake in fungi and this may be a result of fewer studies demonstrating the efficacy of SIGS against phytopathogenic fungi.

## 4. Importance of Understanding dsRNA Uptake

### 4.1. Resistance

Historically speaking, following the deployment of a novel pest or pathogen control measure, strains resistant to the technology eventually emerge [105]. Given that RNAi is a sequence-specific process, changes in a target gene’s sequence could potentially render the pest or pathogen resistant, but this type of resistance can be easily and quickly overcome by changing gene targets or sequences. A more problematic resistance to overcome would be a change in the uptake mechanism. While it seems unlikely that an organism would tolerate substantive changes to essential proteins such as clathrin, it is possible that dsRNA-specific receptors or channels could be modified to no longer function in promoting uptake [106]. Reduced dsRNA uptake through resistance has already been demonstrated in a laboratory strain of corn rootworm (*D. virgifera*) that was exposed to progressively higher doses of dsRNA through several generations [107]. Within eleven generations, insects had greater than a 130-fold increase in resistance. The nature of the resistance was attributed to significantly reduced gut luminal uptake, and the molecular mode behind the mutations was linked to a single recessive locus. The mechanism of which these mutations altered uptake is unknown. Although this resistant strain was developed in a laboratory setting, it highlights that resistance through reduced uptake can indeed occur. Researchers are attempting to minimize this risk by investigating multiple modes in which dsRNA can be delivered to the cells by different vehicles or carriers. If different modes of uptake can be achieved that are not reliant on a single dsRNA specific receptor, then the risk of RNAi insensitivity will be reduced. While much remains to be understood about many of these different carriers, they show considerable promise in using dsRNA-based control methods for a wide range of pests and pathogens. 

### 4.2. dsRNA Formulations

SIGS using naked dsRNA has been shown to be an effective control method against both insect pests and fungal pathogens [31,52,108]. Despite these advances, topically applied dsRNA molecules are vulnerable to degradation. Environmental factors such as water, sunlight/UV light, and interactions with soil microbes all contribute to a reduced availability of dsRNA on plant surfaces and possible absorption into plant tissues [109,110,111]. Upon ingestion by an insect, dsRNA also must contend with dsRNA-degrading enzymes and an unfavorable pH level within the gut [112,113]. To solve these issues, carrier molecules can be utilized to protect the dsRNA, increasing bioavailability and improving control of pathogens and pests [114]. For most carrier particles, a primary goal is to increase stability and persistence of the dsRNA before and after ingestion, to protect the dsRNA from abiotic environmental factors and biotic factors within the insect gut [115,116]. While the dsRNA carriers are often providing some degree of protection from the gut environment of insects, it is important to understand how these carriers interact at the cellular level of uptake, and whether they also play a role in translocating dsRNA into cellular compartments where they can take effect. 

There has been extensive work on human therapeutics on the delivery of dsRNA and siRNA-based treatments through carrier molecules and these may also prove to be valuable to agricultural pest control [117]. While most investigations of dsRNA carriers for plant protection have focused on insects, the enhanced stability and penetrability of some formulations will also be applicable to phytopathogenic fungi. While the barriers of gut pH and nucleases are not an issue for fungi, stability from environmental conditions is still critical. It is difficult to predict when a fungal outbreak is going to occur, and therefore the longer the preventative antifungal treatment can remain intact on the plant surface, the more likely it will be effective when the infection emerges. Additionally, several necrotrophic pathogens, such as *S. sclerotiorum*, can become systemic within the plant in a matter of days [118]. This underlies the importance of getting the optimized load of dsRNA into the fungus as quickly as possible, and carriers enhancing penetrability have the potential to do this.

### 4.3. Nanoparticles

When dsRNA is ingested by an insect, degradation by gut nucleases continues until absorption into the cells occurs. The RNAi effect diminishes the longer the naked dsRNA is exposed to this harsh environment [48]. One of the most commonly used polymers to generate nanoparticles to protect and deliver dsRNA and siRNA to target cells is chitosan [119]. Chitosan is a biodegradable and nontoxic polymer, prepared through the deacetylation of the highly abundant biopolymer chitin [120]. Electrostatic binding between the dsRNA and the chitosan occurs through the negative charges on the phosphate backbone of the dsRNA binding to the positively charged amino group of the chitosan [121]. Chitosan-based formulations have been shown to improve stability from endonucleases and uptake in a number of insect species including *Anopheles gambiae, A. aegypti, Chilo suppressalis,* and *S. frugipera* [121,122,123,124]. Interestingly, chitosan appears to improve uptake in lepidopteran insects such as *S. frugipera,* which are normally inefficient in terms of RNAi response [124]. Naked formulations of dsRNA appear to get trapped in endosomes and are unable to induce silencing, however dsRNA complexed with chitosan showed reduced accumulation within these endosomes. While it is unknown if the dsRNA-chitosan conjugate enters through endocytic mechanisms similar to naked dsRNA or through alternative mechanisms, it is highly encouraging that this formulation can improve efficiency in species that were previously insensitive to RNAi.

Layered double hydroxide clay nanosheets are another nanoparticle delivery system that shows promise in increasing RNAi efficiency in pest control. Stacks of positively charged nanosheets are able to electrostatically bind the negative charges of the dsRNA and provide improved protection against environmental elements and nuclease activity. Mitter et al. [125] showed that atmospheric conditions slowly break down the clay nanosheets on the plant surface releasing the dsRNA. In their study, the clay nanosheet provided protection against pepper mild mottle virus up to 20 days post spraying, providing a much longer window of protection compared to the naked dsRNA treatment. This technology also holds potential to be of utility in insect and fungal protection due to this increased length of bioactivity. Interestingly, this formulation also appears to encourage uptake and systemic spread within the host plant that was sprayed. Mitter et al. [125] were able to detect dsRNA in distal, unsprayed regions of the plant indicating that whole plant protection can be achieved after spraying only a portion of the plant. 

### 4.4. Ribonucleotide Protein dsRNA Carriers

The plasma membrane is the primary barrier of uptake, and dsRNA’s negative charge prevents passive transport through the negatively charged membrane. To remedy this, cell penetrating peptides (CPPs) can facilitate uptake into the epithelial gut cells and provide protection from nucleases. CPPs are a class of peptides able to cross cellular membranes and can function as a carrier for siRNAs, proteins, additional peptides, and other small molecules [126]. More specifically, the cationic, arginine-rich Tat peptides have been shown to successfully internalize plasmid DNA and hormones within insect cells [127,128]. An improved version of this Tat peptide is the peptide transduction domain (PTD), which unloads the carrier’s cargo by destabilizing the vesicle membrane following endocytosis [129]. By pairing the PTD with a dsRNA binding domain (DRBD), ribonucleoprotein particles (RNP) can be formed to carry dsRNA past the plasma membrane, escape the endosome, and induce silencing. Gillet et al. [116] demonstrated that RNPs can improve oral delivery of dsRNA and enhance RNAi effects.

PTD-eGFP applied to a cotton boll weevil (*Anthonomus grandis*) gut cell suspension showed PTD-eGFP clustered at the membrane of gut cells within 2 min and showed colocalization with FM4-64 endosomal stain within vesicles. When *A. grandis* midgut was incubated with Cy3-labeled dsRNA complexed with PTD-DRBD, the particles were shown to associate with the plasma membrane, within endovesicles, and ultimately into the cytoplasm. Transcript knockdown of a chitin synthase gene was improved with oral delivery of the RNPs compared to naked dsRNA, demonstrating the utility of CPPs in delivering dsRNAs to the cytoplasm of insect cells. The PTD peptide used in this study was optimized to carry short dsRNAs or siRNAs [130,131] and may need to be modified to ensure the effective delivery of long dsRNAs typically used in insect RNAi applications. With the research being conducted to overcome negatively charged membranes using cell-penetrating peptides, there may be discoveries that could transfer to fungal-based systems since CPPs have been shown to be effective nanocarriers of other antifungal compounds in fungal systems [132,133].

### 4.5. Cationic Liposome dsRNA Carriers

In pharmacological studies where efficient drug delivery is highly dependent on absorption into cells, lipid carrier molecules have been observed to facilitate gene delivery. Cationic liposomes consist of cationic and neutrally charged lipids that encapsulate nucleic acid to form lipoplexes [134]. The positively charged outer lipid coating allows association with the plasma membrane and isolates the negative charges to inside the liposome. One of the first instances of using a liposome-based delivery method was to encapsulate an antiviral, immunomodulating dsRNA to protect against influenza in mice [135]. Since then, this technology has been applied in insects, fungi, nematodes, and crustacean viruses [136,137,138,139] with successes in lowering gene expression and/or mortality. 

In the limited number of studies conducted so far, interactions between insect gut epithelial cells and lipoplexes have yet to be understood. However, insights from recent papers in other cell types may provide a better understanding of the role lipoplexes have in associating with the plasma membrane and subsequent release into the cytosol. Sarker [140] reported that cationic liposomes containing a fluorescently marked phallotoxin were indeed associated with the plasma membrane of HeLa cancer cells and were translocated via an endocytic pathway. Interestingly the phallotoxin was not released within 30 min of incubation but was released into the periphery of the cell by 24 h. To confirm liposomes were entering via endocytosis, HeLA cells were treated with chemical inhibitors of clathrin-mediated endocytosis (chlorpromazine), caveolae-mediated endocytosis (nystatin), and micropinocytosis (cytochalasin D). Fluorescent confocal imaging appeared to show caveolae-mediated endocytosis is a primary pathway for lipoplexes, but the other two endocytic pathways also had some role in uptake. A similar study [141] in mosquitoes (*A. aegypti*) found caveolae-mediated endocytosis is essential for nanoparticle (anhydride) internalization into epithelial cells. This is especially true for smaller particles (<100 nm) but could also allow larger particles such as lipoplexes which can easily exceed that size. Pharmacological studies have also confirmed that dsRNA lipoplexes are an effective mode of treatment against the human fungal pathogen *Aspergillus flavus,* however no mode of uptake was described [137]. Chavan et al. [142] showed that liposomes have an affinity for the *β*-glucan in *Aspergillus* cell walls, which is primarily exposed at the septa of the hyphae during growth and division of the cells. Given that lipoplexes appear to deliver dsRNA by a mechanism other than clathrin-mediated endocytosis, they could potentially prove effective in administering dsRNAs to RNAi insensitive organisms such as the lepidopterans, as well as strains resistant to clathrin-mediated uptake. 

### 4.6. Concluding Remarks

RNAi technologies hold the potential to generate a novel class of pesticides to provide growers with additional tools to overcome unpredictable changes and guidelines to traditional chemistries. Despite recent advances in dsRNA uptake mechanisms in insect species, much remains to be discovered. In fungi, even less is known, where the formulation, uptake, and processing of dsRNAs remains relatively undescribed. Studying the durability and delivery methods of dsRNAs, and more specifically the uptake of these dsRNAs into the target organism remains ripe for investigation. As with any crop protection strategy, resistance is always a concern. Uptake mechanisms will shine a light on potential areas where resistance may develop. Ultimately, the answers to these questions will play an important role in the successful implementation of RNAi in agriculture. The development of successful dsRNA formulations that protect, facilitate delivery, and discourage resistance buildup, will guarantee RNAi as the next generation of crop protection tools for improved agricultural outputs.
